# Global market trends of flavor capsule cigarettes and menthol (non-capsule) cigarettes: An ecological analysis using commercial data across 78 countries, 2010–2020

**DOI:** 10.18332/tid/153974

**Published:** 2022-10-10

**Authors:** Christina N. Kyriakos, Dickson Qi, Kiara Chang, Anthony A. Laverty, Filippos T. Filippidis

**Affiliations:** 1 Public Health Policy Evaluation Unit, Department of Primary Care and Public Health, School of Public Health, Imperial College London, London, United Kingdom

**Keywords:** cigarette market share, menthol, flavor capsule cigarettes, global tobacco control

## Abstract

**INTRODUCTION:**

This study describes market trends of flavor capsule cigarettes (FCCs) and menthol (non-capsule) cigarettes (MNCCs) across 78 countries from 2010 to 2020 and examines country-level factors associated with market shares of these products.

**METHODS:**

Market share and retail volume data came from the Euromonitor Passport database and country-level data came from the World Health Organization (WHO), World Bank, and International Monetary Fund. Multivariable linear fixed effects panel regression analyses were conducted to evaluate the relationship between predictor variables and the market shares of menthol (non-capsule) cigarettes and flavor capsule cigarettes.

**RESULTS:**

The overall market share (i.e. the percentage retail volume out of total retail volume of all cigarette types) increased from 0.23% in 2010 to 4.5% in 2020 for FCCs and decreased from 5.0% to 3.8% for MNCCs. Market shares of FCCs grew most rapidly in the Americas region and among upper middle-income countries. Market shares of MNCCs remained stable across most regions and were highest in the Western Pacific and Africa regions. The overall market share of FCCs was positively associated with the unemployment rate (β=0.28; 95% CI: 0.12–0.44, p=0.001), and inversely associated with the percent of the population aged 15–29 years (β= -0.57, 95% CI: -0.98 – -0.15, p=0.008), percent of urban population (β= -0.88; 95% CI: -1.28 – -0.48, p<0.001), GDP PPP per capita (β= -0.13; 95% CI: -0.24 – -0.03, p=0.015), and age-standardized prevalence of cigarette smoking (β= -0.93; 95% CI: -1.38 – -0.49, p<0.001). In contrast, the overall market share of MNCCs was positively associated with urbanicity (β=0.24; 95% CI: 0.08–0.40, p=0.003), and negatively associated with the unemployment rate (β= -0.09; 95% CI: -0.17 – -0.02, p=0.014).

**CONCLUSIONS:**

Global sales of flavor capsule cigarettes grew substantially in the last decade, surpassing menthol (non-capsule) cigarettes, which also continued to be high in many regions. There is a need for increased efforts to address flavors and novel tobacco products, features that are known to appeal to youth.

## INTRODUCTION

Tobacco use continues to threaten public health as a leading cause of preventable morbidity and mortality worldwide^1^. In an effort to address the global burden of tobacco use, following the adoption of the World Health Organization (WHO) Framework Convention on Tobacco Control (FCTC), the first international health treaty^2^, the WHO established six MPOWER measures to help countries implement effective interventions proven to reduce tobacco demand^2^. These measures include: **M**onitor tobacco use and prevention policies; **P**rotect people from tobacco smoke; **O**ffer help to quit tobacco use; **W**arn about the dangers of tobacco and anti-tobacco mass media campaigns; **E**nforce bans on tobacco advertising, promotion and sponsorship; and **R**aise taxes on tobacco.

With countries making progress towards implementing these tobacco control measures^3^, the tobacco industry has responded in new ways to promote their products. Innovation has emerged as a key marketing strategy of transnational tobacco companies^4^. Historically, the tobacco industry has added flavorings, including the most common flavor menthol, to tobacco products^5^. Evidence suggests that flavors encourage smoking initiation and progression to regular use, particularly through their role in increasing palatability and appeal of tobacco^5–8^. Moreover, menthol’s cooling and anesthetic properties can reduce the harshness of tobacco smoke on the throat, thereby facilitating inhalation^5^. Tobacco industry marketing of MNCCs to various targeted populations, such as adolescents, females, and racial/ethnic and sexual minority groups, is well documented^5,9,10^. More recently, since 2007, flavor capsule cigarettes (FCCs), which contain one or more capsules in the filter that release flavoring when crushed by the consumer, have entered the market^11^. Advertised by the tobacco industry as being technologically advanced, offering consumer choice/ customization and a unique sensory experience, FCCs have gained popularity globally. In some countries FCCs only come in menthol flavor, while in others a plethora of flavors are available on the market^12^. While there is overlap in these product groups, global market trends may differ between MNCCs and FCCs.

Monitoring global tobacco use, including surveillance of market trends, is critical for identifying needs and priority areas for increased tobacco prevention and control strategies, and aligns with the M of the MPOWER measures (Monitor). However, studies examining MNCC market data are largely concentrated in specific countries, such as the United States^13-15^, Australia^16^, and Singapore^17^. Global data on MNCC market shares are limited and/or outdated. The most comprehensive global study on MNCC market shares, which covers 55 countries, dates back to 2001^18^. Some studies have used the commercial database Euromonitor Passport to explore short-term trends in global market shares of FCCs. These have highlighted that the largest FCC markets are in Latin American countries, including Peru, Guatemala, Mexico, and Argentina, where market shares increased by at least 40% between 2014 and 2017^11,19^. Another study presents Euromonitor data on combined market shares of MNCCs and FCCs, across the UK and the EU member states, and other countries in the WHO Europe Region, demonstrating a growth in sales from 2004 to 2018. The EU, however, has shown a downward trend from 2015, after the EU/UK characterizing flavor ban was announced^20^. Another study examined combined menthol and capsule cigarette market trends by country income level using Euromonitor data^21^. This study found market growth from 2005 to 2019 in both upper middle-income (4.0% to 12.3%) and lower middle-income (2.5% to 6.5%) countries (with no market data for low-income countries)^21^. While they are important studies, they do not provide comprehensive global coverage and/or do not address potential differences in market shares of FCCs and MNCCs.

Several studies have examined individual-level correlates of MNCC and FCC use. Younger age and being female are consistently associated with use of both across many countries, while other characteristics, such as socioeconomic status, race/ethnicity, and living in rural areas, generally vary by country or findings are mixed^5,18,22-25^. Little is known, however, about country-level factors that may be related to greater use of these products in some countries over others. Studies suggest that tobacco industry marketing plays a large role in the population use of MNCCs as well as FCCs^12,26^. It is possible, though, that other country-level factors, including policy-related factors, may be driving market share differences. Ecological analyses can provide such insight and may inform tobacco control strategies. Previous studies have examined ecological factors, such as socio-economic factors (Gross Domestic Product^27^, unemployment rate^27,28^), cigarette smoking prevalence^27^, and implementation of MPOWER measures^27,28^, on country-level tobacco-related outcomes (e.g. prevalence of electronic cigarette use^27^; tobacco policy implementation^28^). Similar factors may be associated with market trends of MNCCs and FCCs.

Given these research gaps, the aims of this ecological study were to describe trends in global market shares of MNCCs and FCCs, and to assess associations with country-level factors across 78 countries from 2010 to 2020.

## METHODS

### Data sources and measures

*Euromonitor passport market data*

Market data come from Euromonitor Passport, an online database by the market research company Euromonitor International, which collects market data from several retail sources^29^. This dataset, has been previously used for tobacco control research^30-32^ and can serve to inform epidemiological surveillance. Data were available for 78 countries, none of which was low-income. Annual data collected between 2010 and 2020 for available countries obtained from Euromonitor included total retail volume (in million sticks) and percent market shares for FCCs (including menthol flavor) and MNCCs. The overall percent market shares of FCCs and MNCCs were calculated by Euromonitor as their respective proportions out of the total retail volume of cigarettes of all types (i.e. FCCs, MNCCs, standard cigarettes).

*Country-level sociodemographic characteristics*

Countries were categorized according to their geographical regions as defined by the WHO: African, Eastern Mediterranean, European, Americas, South-East Asia, and Western Pacific^33^. Income level classification also came from the World Bank and is based on Gross National Income (GNI) per capita (Atlas method, current US$) in 2020 (classified as of 1st July 2021, adjusted for inflation): low-income countries (<$1046), lower middle-income countries ($1046–4095), upper middle-income countries ($4096–12695), and high-income countries (>$12695)^34^.

The following sociodemographic data were also obtained from the World Bank: male and female population as percent of total population, male population aged 15-29 as percent of total male population, female population aged 15–29 years as percent of total female population, urban population as percent of total population, total unemployment as percent of total labor force. We derived the percent of population aged 15–29 years using the sum of female population aged 15–29 years and male population aged 15–29 years then divided by the total population. Gross Domestic Product (GDP) purchasing power parity (PPP) in international dollars per capita was obtained from the International Monetary Fund World Economic Outlook Database^35^. We derived GDP PPP in thousands of international dollars per capita by dividing by 1000.

*Country-level smoking prevalence and MPOWER implementation*

Country-level age-standardized estimates of cigarette smoking prevalence (as % of adult population aged ≥15 years) and data on country-level implementation of MPOWER were obtained from the WHO Global Health Observatory on Tobacco Control^36^. Data were available for the years 2010, 2012, 2014, 2016 and 2018, hence we used these to interpolate for the years 2011, 2013, 2015, 2017, and to extrapolate for 2019 and 2020. MPOWER measures include: **M**onitor tobacco use and prevention policies; **P**rotect people from tobacco smoke; **O**ffer help to quit tobacco use; **W**arn about the dangers of tobacco and anti-tobacco mass media campaigns; **E**nforce bans on tobacco advertising, promotion and sponsorship; and **R**aise taxes on tobacco. A score of 1 represents lack of data and scores 2–4 for M and 2–5 for POWER indicate increasing levels of implementation. A total MPOWER score was calculated by summing individual measure scores, ranging from a minimum of 6 (1 in each of the six measure scores) to a maximum of 29 (4 in M score and 5 in POWER scores)^37^.

### Statistical analysis

*Descriptive analyses*

Country-level characteristics are presented as medians with interquartile range (IQR). We further present country-level mean values by WHO geographical region and World Bank country income groups. We derived retail volume per capita for the FCCs and MNCCs using the respective retail volume divided by the country’s total population size. We calculated annual growth rates (AGRs) in FCC and MNCC market shares for each country using the respective percent market shares from consecutive years, with the following formula for year *n*:


$$AGR\;of\;year_{n}\text{ = }\left(Market\;share\;of\;year_{n}\;-\;Market\;share\;of\;year_{n-1}\right)/Market\;share\;of\;year_{n-1}.$$

AGR values above 300% were capped at 300%, to avoid meaningless values resulting from an increase over a very small market share. We calculated average annual growth rates (AAGRs) of the FCC and MNCC market shares for each country were calculated as:


$$AAGR=(\frac{1}{N})\Sigma_{n=1}^{N}AGR_{n}$$

where *N* is the total number of years. We plotted country-level AAGR from 2010 to 2020 against percent market share for 2020 (or the latest available year) for FCCs and MNCCs, using bubble charts, with the size of the bubble representing the total cigarette retail volume.

*Panel regression analysis*

Linear fixed effects panel regression analyses were performed to evaluate the relationship between changes in country-level factors described above and changes in country-level percent market shares of FCCs and MNCCs, using Stata/SE 16.1. Fixed effects models were considered more appropriate than random effects models, as supported by the results of Hausman tests which showed a p<0.05. Also, fixed effects model controls for all time-invariant factors and therefore its estimated changes in market outcome will not be biased by the variation in time-invariant factors between countries. We examined predictor variables that have been found to be associated with FCC and MNCC use at the individual level, as well as country-level variables that have been associated with other tobacco-related outcomes^5,12,18,22-25,27,28^. Predictor variables were added into the model in incremental steps and no major changes in the magnitude or significance of coefficients were observed during this model fitting process. Presence of multicollinearity were examined by the variance inflation factor among predictor variables but was not identified. Regression coefficients (β) with 95% confidence intervals (CIs) for all predictor variables are presented.

## RESULTS

### Overall market trends of FCCs and MNCCs from 2010 to 2020

**Figure 1:**
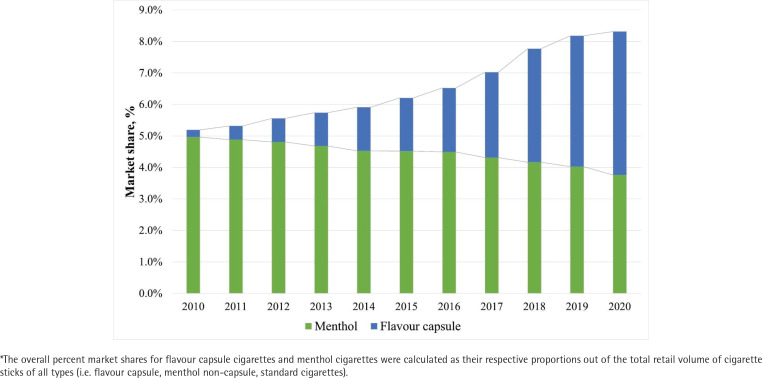
Overall percent market share* of flavor capsule cigarettes and menthol (non-capsule) cigarettes from 2010 to 2020 across 78 countries, Euromonitor Passport

Medians with IQR of country-level characteristics and market outcomes in 2010 and in 2020 are presented in Table 1. The median percent market shares across countries for FCCs and MNCCs were 0.5% (IQR: 0.2–1.8; n=21 countries) and 4.1% (IQR: 1.7–10.6; n=78) in 2010 and 3.3% (IQR: 1.2–8.5, n=64) and 2.4% (IQR: 0.8–6.8; n=74) in 2020. The median retail volumes per capita of FCCs and MNCCs were 2.8 (IQR: 1.9–13.7) and 32.6 (IQR: 12.8–72.5) in 2010 and 18.7 (IQR: 4.7–48.9) and 14.6 (IQR: 4.0–33.7) in 2020. As depicted in Figure 1, the overall percent market share (i.e. total retail volume out of all cigarette types) increased from 0.2% in 2010 to 4.5% in 2020 for FCCs, surpassing MNCCs which decreased from 5.0% to 3.8% by 2019.

**Table 1:** Median values of country-level characteristics and market outcomes in 2010 and 2020

	*2010*			*2020*		
	*n*	*Median*	*IQR*	*n*	*Median*	*IQR*
**Characteristics**						
**Sociodemographic**						
Female population (% of total population)^a^	77	50.7	50.0–51.3	77	50.6	50.0–51.3
Population aged 15–29 years (% of total population)^a^	77	22.5	19.6–27.4	77	19.2	16.8–23.7
Urban population (% of total population)^a^	77	68.9	55.2–80.9	77	73.7	57.7–82.7
Unemployment rate (% of total work force)^a^	77	7.3	4.8–10.0	77	6.2	4.7–9.3
GDP per capita purchasing power parity^b^						
(International dollars, in thousands)	78	18.6	10.5–36.4	78	27.9	13.3–45.3
**Smoking^c^**						
Age-standardized prevalence of cigarette smoking (%)*	71	23.5	16.2–28.7	71	19.8	12.0–25.3
MPOWER overall score (range: 6–29)	75	21.0	18.5–22.5	75	24.0	21.5–25.5
**Market outcomes by cigarette flavor category^d^**						
**Flavored capsule cigarettes**	21			64		
Market share (% retail volume)		0.5	0.2–1.8		3.3	1.2–8.5
Retail volume (in million sticks)		84.6	15.4–264.9		228.2	69.4–2410.8
Retail volume per capita		2.8	1.9–13.7		18.7	4.7–48.9
**Menthol (non-capsule) cigarettes**	78			74		
Market share (% retail volume)		4.1	1.7–10.6		2.4	0.8–6.8
Retail volume (in million sticks)		597.8	185.6–1459.5		225.5	68.1–1014.2
Retail volume per capita		32.6	12.8–72.5		14.6	4.0–33.7
**Standard cigarettes**	78			78		
Market share (% retail volume)		95.7	89.4–98.3		93.4	79.9–98.0
Retail volume (in million sticks)		13668.7	5074.7–43301.4		9883.0	3689.0–30937.1
Retail volume per capita		903.9	451.8–1446.0		704.7	257.8–1055.5
**Total cigarettes**	78			78		
Market share (% retail volume)		100.0				
Retail volume (in million sticks)		14696.2	5098.3–46736.5		11238.5	3751.3–35801.5
Retail volume per capita		985.0	493.6–1532.4		751.6	366.6–1106.8

* Age-standardized prevalence of cigarette smoking in 2020 was projected by the WHO Global Health Observatory from data of previous years.

a World Bank,

b International Monetary Fund,

c WHO Global Health Observatory,

d Euromonitor Passport. IQR: interquartile range.

### Market trends of FCCs by WHO region and country income level

**Figure 2:**
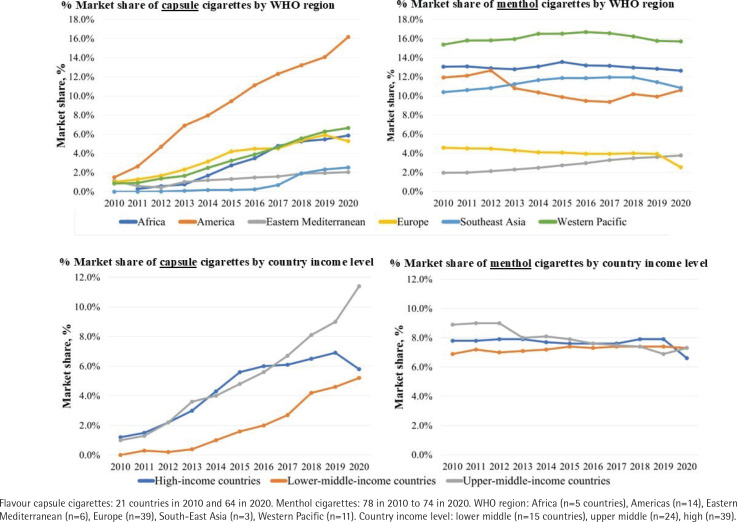
Average percent market share of flavour capsule cigarettes and menthol cigarettes by WHO region and country income level from 2010 to 2020, Euromonitor Passport

The average market share of FCCs generally increased across all WHO regions from 2010 to 2020, with the exception of Europe which saw an increase until 2019, followed by a decrease of 0.64 percentage points from 2019 to 2020 (Figure 2). Market increases in the region of the Americas were greater than other WHO regions (from 1.5% to 16.2%) (Table 2). This was mainly driven by the high market growth in Latin American countries, which made up four of the top five countries with the highest FCC market shares in 2020: Chile (48.4%), Peru (35.0%), Guatemala (32.6%), Mexico (27.3%), and South Korea (24.7%) (Table 3). There was an overall increase in FCC market share for lower middle-income, upper middle-income, and high-income country groups from 2010 to 2020 (Figure 2). The greatest increase was experienced by upper middle-income countries; on average each country’s market share increased by 9.93 percentage points during 2010–2020. These upward trends are further depicted in Supplementary file Figure 1, where the AAGR from 2010 to 2020 for FCCs was positive for the majority of countries (n=67), with the exception of five EU European countries. Four of the top five countries with highest AAGRs were lower and upper middle-income countries, three of which were non-EU European countries, including: India (154.1%), Uzbekistan (122.5%), Uruguay (114.8%), Russia (84.3%), and Ukraine (84.2%).

**Table 2:** Average market share (%) of flavor capsule cigarettes and menthol cigarettes from 2010 to 2020 by WHO region and country income level, Euromonitor Passport (for n countries in each region)

		*WHO Region*						*Country income level*		
*Year*	*Overall*	*Africa (n=5)*	*Americas (n=14)*	*Eastern Mediterranean (n=6)*	*Europe (n=39)*	*South-East Asia (n=3)*	*Western Pacific (n=11)*	*High (n=39)*	*Upper middle (n=24)*	*Lower middle (n=15)*
	*%*	*%*	*%*	*%*	*%*	*%*	*%*	*%*	*%*	*%*
**Flavor capsule cigarettes**										
2010	1.1	-	1.5	1.1	1.0	0.0	0.9	1.2	1.0	0.0
2011	1.4	0.3	2.6	0.6	1.3	0.0	0.9	1.5	1.3	0.3
2012	2.0	0.6	4.7	0.4	1.7	0.0	1.4	2.2	2.2	0.2
2013	2.8	0.8	6.9	1.0	2.3	0.1	1.7	3.0	3.6	0.4
2014	3.7	1.7	8.0	1.2	3.1	0.2	2.5	4.3	4.0	1.0
2015	4.7	2.7	9.5	1.3	4.2	0.2	3.2	5.6	4.8	1.6
2016	5.2	3.5	11.1	1.5	4.5	0.2	3.9	6.0	5.6	2.0
2017	5.7	4.8	12.3	1.6	4.5	0.7	4.7	6.1	6.7	2.7
2018	6.6	5.3	13.2	1.9	5.4	1.9	5.6	6.5	8.1	4.2
2019	7.1	5.5	14.1	2.0	5.9	2.3	6.3	6.9	9.0	4.6
2020	7.4	5.9	16.2	2.0	5.3	2.5	6.7	5.8	11.4	5.2
**Menthol (non-capsule) cigarettes**										
2010	8.0	13.1	11.9	2.0	4.6	10.4	15.4	7.8	8.9	6.9
2011	8.1	13.1	12.1	2.0	4.5	10.6	15.8	7.8	9.0	7.2
2012	8.1	12.9	12.7	2.1	4.5	10.8	15.8	7.9	9.0	7.0
2013	7.8	12.8	10.8	2.3	4.3	11.2	16.0	7.9	8.0	7.1
2014	7.7	13.1	10.4	2.5	4.1	11.7	16.5	7.7	8.1	7.2
2015	7.7	13.6	9.9	2.7	4.1	11.9	16.5	7.6	7.9	7.4
2016	7.6	13.2	9.5	3.0	4.0	11.9	16.7	7.6	7.6	7.3
2017	7.5	13.2	9.4	3.3	3.9	11.9	16.6	7.6	7.5	7.4
2018	7.6	13.0	10.2	3.5	4.0	11.9	16.2	7.9	7.4	7.4
2019	7.5	12.8	9.9	3.6	3.9	11.4	15.8	7.9	6.9	7.4
2020	6.9	12.6	10.6	3.8	2.5	10.8	15.7	6.6	7.3	7.3

**Table 3:** Market share and average annual growth rate (AAGR) for flavor capsule cigarettes and menthol cigarettes by country (N=78), Euromonitor Passport 2010–2020

			*Flavor capsule cigarettes*		*Menthol (non-capsule) cigarettes*	
*Country*	*WHO geographical region*	*World Bank country income level (2020)*	% Market share*	*% AAGR*	% Market share*	*% AAGR*
Algeria	Africa	Lower middle	0.6	5.9	0.4	5.0
Argentina	Americas	Upper middle	20.0	47.3	0.1	-9.5
Australia	Western Pacific	High	2.7	6.5	6.8	-0.7
Austria	Europe	High	1.5	30.5	0.2	-19.3
Azerbaijan	Europe	Upper middle	5.8	33.0	2.2	-7.1
Belarus	Europe	Upper middle	19.3	83.5	1.5	25.4
Belgium	Europe	High	-	-	1.9	-6.3
Bolivia	Americas	Lower middle	1.6	61.3	5.2	-1.0
Bosnia and Herzegovina	Europe	Upper middle	1.7	51.3	0.0	14.9
Brazil	Americas	Upper middle	3.9	57.0	2.8	-12.2
Bulgaria	Europe	Upper middle	0.0 (2017)	-13.2	0.5 (2019)	-11.7
Cameroon	Africa	Lower middle	0.5	15.2	29.9	3.7
Canada	Americas	High	-	-	0.6 (2017)	-14.5
Chile	Americas	High	48.4	38.0	6.0	38.7
China	Western Pacific	Upper middle	2.8	73.6	0.1	2.9
Colombia	Americas	Upper middle	1.2	8.0	21.9	1.8
Costa Rica	Americas	Upper middle	18.2	26.5	1.0 (2019)	-11.2
Croatia	Europe	High	1.2	24.3	0.1	-11.3
Czech Republic	Europe	High	3.6 (2017)	17.4	6.5	5.0
Denmark	Europe	High	1.9	41.1	2.8	-4.7
Dominican Republic	Americas	Upper middle	2.5	34.1	32.7	2.6
Ecuador	Americas	Upper middle	5.4	37.7	1.2	-10.1
Egypt	Eastern Mediterranean	Lower middle	2.3	67.4	0.0	6.3
Estonia	Europe	High	0.4	19.7	0.4	-16.2
Finland	Europe	High	6.0 (2016)	35.4	5.8	-9.6
France	Europe	High	3.6 (2016)	70.4	1.4	-7.8
Georgia	Europe	Upper middle	2.6	13.2	3.2	-2.6
Germany	Europe	High	-	-	0.8	-5.8
Greece	Europe	High	0.2	7.8	0.1	17.4
Guatemala	Americas	Upper middle	32.6	37.7	9.6	-4.1
Hong Kong	Western Pacific	High	13.3	81.2	27.3	-0.2
Hungary	Europe	High	12.1 (2017)	52.5	8.1	6.7
India	South-East Asia	Lower middle	6.3	154.1	0.8	6.2
Indonesia	South-East Asia	Lower middle	0.5	60.0	4.4	10.0
Ireland	Europe	High	1.3	48.4	0.0	-16.4
Israel	Europe	High	0.0	41.8	1.0	-6.2
Italy	Europe	High	0.0	-8.2	0.1	-10.1
Japan	Western Pacific	High	7.0	15.9	27.7	2.9
Kazakhstan	Europe	Upper middle	16.8	54.6	0.7	-15.3
Kenya	Africa	Lower middle	0.5	6.4	5.9	2.8
Latvia	Europe	High	4.7	-0.2	1.5	-2.9
Lithuania	Europe	High	3.9	29.3	3.9	-1.0
Malaysia	Western Pacific	Upper middle	0.7	20.5	24.8	1.3
Mexico	Americas	Upper middle	27.3	13.0	0.4	-26.4
Morocco	Eastern Mediterranean	Lower middle	0.6	14.1	4.2	3.7
Netherlands	Europe	High	0.6 (2017)	12.8	1.4	-10.1
New Zealand	Western Pacific	High	4.7	48.7	9.6	-1.0
Nigeria	Africa	Lower middle	18.7	75.5	24.1	-2.4
North Macedonia	Europe	Upper middle	-	-	0.0	0.9
Norway	Europe	High	6.4	14.1	9.1	-0.6
Pakistan	Eastern Mediterranean	Lower middle	-	-	2.7	2.1
Peru	Americas	Upper middle	35.0	39.2	18.0	-2.3
Philippines	Western Pacific	Lower middle	4.6	11.4	22.6	-0.4
Poland	Europe	High	7.7	14.2	20.6	3.1
Portugal	Europe	High	6.2	13.5	2.1	-4.4
Romania	Europe	Upper middle	1.9 (2019)	-1.9	2.0	-10.3
Russia	Europe	Upper middle	22.9	84.3	1.8	-6.0
Saudi Arabia	Eastern Mediterranean	High	3.4	11.9	12.4	18.9
Serbia	Europe	Upper middle	0.2	19.9	0.2	-2.1
Singapore	Western Pacific	High	3.2	41.2	47.5	0.1
Slovakia	Europe	High	2.9	45.1	1.0	-17.7
Slovenia	Europe	High	1.3	-6.8	0.7	-7.8
South Africa	Africa	Upper middle	9.0	36.9	2.9	-9.7
South Korea	Western Pacific	High	24.7	60.6	2.8	-4.7
Spain	Europe	High	0.3	21.7	0.0	-21.3
Sweden	Europe	High	1.8	50.0	4.2	-10.2
Switzerland	Europe	High	1.8	31.0	2.4	2.1
Taiwan	Western Pacific	High	8.3	61.7	1.2	2.9
Thailand	South-East Asia	Upper middle	0.8	10.9	27.3	-0.5
Tunisia	Eastern Mediterranean	Lower middle	-	-	3.3	-1.3
Turkey	Europe	Upper middle	0.8 (2019)	38.4	0.3 (2019)	-12.4
Ukraine	Europe	Lower middle	14.8	84.2	2.2	17.3
United Arab	Eastern Mediterranean	High	1.9	49.8	0.1	-10.6
Emirates						
United Kingdom	Europe	High	4.6	69.8	2.4	-6.3
Uruguay	Americas	High	8.7	114.8	0.0	-8.9
USA	Americas	High	5.5	12.3	29.5	0.0
Uzbekistan	Europe	Lower middle	15.7	122.5	1.8	-5.6
Vietnam	Western Pacific	Lower middle	1.2	49.4	2.4	5.5

* 2020 or latest available year.

### Market trends of MNCCs by WHO region and country income level

In contrast, average market shares for MNCCs remained relatively stable from 2010 to 2020 in WHO regions with the highest market shares: Western Pacific (15.4% to 15.7%), Africa (13.1% to 12.6%), Americas (11.9% to 10.6%), and South-East Asia (10.4% and 10.8%) (Figure 2 and Table 2). Average MNCC market shares in the Eastern Mediterranean were comparatively lower, however, they experienced growth from 2.0% in 2010 to 3.8% in 2020, while in Europe market shares decreased over time, with the largest decline from 2019 to 2020. Average market share of MNCCs did not vary by country income level. The five highest MNCC markets in terms of market share in 2020 were: Singapore (47.5%), Dominican Republic (32.7%), Cameroon (29.9%), USA (29.5%), and Japan (27.7%) (Table 3). The majority of countries (n=50) had a negative AAGR for MNCCs (Supplementary file Figure 1). Countries with the highest AAGR included: Chile (38.7%), Belarus (25.4%), Saudi Arabia (18.9%), Greece (17.4%) and Ukraine (17.3%) (Table 3).

### Country-level factors associated with FCC and MNCC market shares

Associations between country-level factors and average percent market shares of FCCs and MNCCs are presented in Table 4. Market share of FCCs increased on average by 0.68 percentage points (95% CI: 0.38 – 0.98) per year from 2010 to 2020 (p<0.001). The following factors were negatively associated with market share of FCCs: percent of the population aged 15–29 years (β= -0.57; 95% CI: -0.98 – -0.15, p=0.008), percent of urban population (β= -0.88; 95% CI: -1.28 – -0.48, p<0.001), GDP PPP per capita (β= -0.13; 95% CI: -0.24 – -0.03, p=0.015), and age-standardized prevalence of cigarette smoking (β= -0.93; 95% CI: -1.38 – -0.49, p<0.001). Unemployment rate was positively associated with market share of FCCs (β=0.28; 95% CI: 0.12–0.44, p=0.001), but no significant association was observed with percent female population and overall MPOWER score.

**Table 4:** Country-level factors associated with average percent market share of flavor capsule cigarettes and menthol cigarettes, from 2010 to 2020, Euromonitor Passport

*Factors*	*Flavor capsule*			*Menthol (non-capsule)*		
	β*	*95% CI*	*p*	β*	*95% CI*	*p*
Year	0.68	0.38 – 0.98	**<0.001**	-0.19	-0.31 – -0.06	**0.003**
Female population (% of total population)	0.34	-0.73 – 1.41	0.530	-0.16	-0.63 – 0.31	0.512
Population aged 15–29 years (% of total population)	-0.57	-0.98 – -0.15	**0.008**	-0.07	-0.23 – 0.10	0.439
Urban population (% of total population)	-0.88	-1.28 – -0.48	**<0.001**	0.24	0.08 – 0.40	**0.003**
Unemployment rate (% of total work force)	0.28	0.12 – 0.44	**0.001**	-0.09	-0.17 – -0.02	**0.014**
GDP PPP per capita (international dollars, in thousands)	-0.13	-0.24 – -0.03	**0.015**	-0.03	-0.08 – 0.01	0.160
Age-standardized prevalence of cigarette smoking	-0.93	-1.38 – -0.49	**<0.001**	-0.14	-0.31 – 0.04	0.124
Overall MPOWER score	0.04	-0.17 – 0.25	0.728	-0.07	-0.16 – 0.03	0.173

Flavor capsule cigarettes: 21 countries in 2010 and 64 in 2020. Menthol cigarettes: 78 countries in 2010 and 74 in 2020.

* Fixed-effects panel linear regression models adjusted for all predictor variables listed in the table. GDP: Gross Domestic Product. PPP: Purchasing Power Parity.

In contrast, market share of MNCCs decreased on average by 0.19 percentage points (95% CI: -0.31 – -0.06) per year from 2010 to 2020 (p=0.003). The percent of urban population was positively associated with market share of MNCCs (β=0.24; 95% CI: 0.08–0.40, p=0.003), while unemployment rate was negatively associated with market share of MNCCs (β= -0.09; 95% CI: -0.17 – -0.02, p=0.014). No other associations were statistically significant.

## DISCUSSION

This study of data from 78 countries found that between 2010 and 2020, global market share of FCCs (including menthol flavor) increased by an average of 0.7% per year, while MNCC share slightly decreased by an average of -0.2% per year. As of 2019, the overall cigarette market share of FCCs surpassed that of MNCCs. The largest increases in FCCs were observed among upper middle-income countries and countries in the WHO Americas region. Average market share of MNCCs remained highest in the WHO Western Pacific and Africa regions. After adjusting for country-level factors, market share of FCCs was positively associated with unemployment rate and negatively associated with percent population aged 15–29 years, percent urban population, GDP PPP, and smoking prevalence. In contrast, the MNCC market share was negatively associated with unemployment rate and positively associated with percent urban population. Neither FCC nor MNCC market shares were associated with percent of female population or overall MPOWER score.

Results from this study add to previous evidence that FCCs have expanded quickly in the past decade^11,19^, with growth continuing as of 2020. Unlike previous studies that have only focused on market share, we also examined retail volume per capita and AAGR, which provide a more nuanced view of FCC trends. While the overall global market share of MNCCs decreased slightly from 2010 to 2020, trends were relatively stable across all WHO regions and country income groups. The overall global decline may not reflect actual reductions in MNCC use because many FCCs are mentholated, and in some countries exclusively so^20^. A study in the US found that while MNCC consumption declined from 2008 to 2020, menthol FCC consumption increased^38^. While this differentiation could not be assessed in the current study, our findings nevertheless indicate a lack of progress in reducing menthol use over the past two decades, particularly among countries with the highest MNCC market shares. Data from 2001 show that the countries among the top five leading MNCC markets included Singapore, Cameroon, and the USA, which continued to be the case in our study in 2020^18^. This is disconcerting given the well-established evidence of the role of menthol in increasing appeal of cigarettes and facilitating smoking initiation and progression to regular use^6,7.^

While an increasing number of countries have adopted flavor cigarette bans^39^, in line with the WHO FCTC Article 9 recommendation to prohibit or restrict ingredients, including flavors^40^, implementation of this measure remains subpar^3^. This, coupled with industry marketing strategies is likely allowing for global market growth of flavored tobacco products^12,41^. The notable market decreases in FCCs and MNCCs in the WHO Europe region from 2019 and 2020 may be partly attributable to implementation of the EU ban on characterizing flavors in cigarettes, which took effect in May 2017, with the exception of menthol, which had a grace period until May 2020.

Our finding, that overall MPOWER scores were not associated with either FCC or MNCC subpar market shares, may at least in part be due to the fact that these measures do not account for implementation of WHO FCTC Article 9 on regulating the content of tobacco products. Moreover, the rise in flavored tobacco products has occurred even in some countries with the highest levels of WHO FCTC implementation, such as in Latin America^42^. However, to date no Latin American country has successfully implemented a ban on MNCCs^43^. While Brazil was the first country worldwide to adopt a ban on all flavor additives, including menthol, in 2012, policy implementation continues to be held up by tobacco industry litigation^44^. Many Latin American countries have also experienced a decrease in the overall smoking prevalence of cigarettes over time^42^, which may explain the inverse association found between smoking prevalence and FCC market share. It is possible that the tobacco industry is concentrating efforts where smoking prevalence is declining to increase product use. There is also evidence that the tobacco industry has used innovations such as FCCs as a marketing strategy to drive sales, particularly in light of marketing restrictions and other tobacco control policies^4,12^. For instance, adoption of standardized packaging legislation in Australia, Singapore and the UK was followed by the introduction of several new FCC products to the market^12,45-47^. It is therefore not surprising that FCCs are often marketed using less regulated avenues, such as advertising at the point-of-sale and the use of packaging, which has particularly been observed in Latin American countries and in low- and middle-income countries, which as indicated in our study is where FCC use is highest^12,21^. In many Latin American countries, retail availability of FCCs, often near schools, is ubiquitous^48-51^.

The rapid rise of these products is alarming given that FCCs are particularly popular among youth and young adults, as well as females in many countries^11,19,20,23^. However, we found that at a country level, market trends were not associated with the percent of population that are female and were in fact inversely associated with the percent of young people aged 15–29 years in the population. At an ecological level, these factors may not change enough over time to explain trends in market shares. Our additional findings that unemployment rate, urbanicity, GDP PPP per capita were associated with the MNCC and/or FCC market share may explain factors driving market variations of these products across different countries. It is also plausible though that growth of these products in some regions over others was not primarily driven by country-level characteristics, but rather a by-product of tobacco industry marketing priorities. While market share of FCCs was positively associated with unemployment rate and negatively associated with percent urban population, the inverse was found for the MNCC market share, which may be reflective of different industry marketing strategies for the respective products.

### Strengths and limitations

There are limitations of this study that must be considered. Euromonitor International has been collaborating with the tobacco industry in recent years. We do not have reason to believe that this affected the quality of market data that we have used, unlike other types of data (e.g. illicit trade), although their methodology was not documented in detail^52,53^. The absence of data from low-income countries limits generalizability of findings, particularly in analyses assessing associations between country income level and market outcomes. However, the included countries had a combined population of 6.21 billion in 2020, accounting for 80.1% of the world population. In addition, we interpolated and extrapolated years of data for prevalence of cigarette smoking and MPOWER measures in order to preserve granularity in the annual market outcomes obtained, which may have attenuated associations between variables. In this study, FCCs and MNCCs were examined as separate categories. However, many capsule cigarettes are menthol flavored and therefore the market of MNCCs may be underestimated in these analyses. Despite these limitations, this study is strengthened by the ability to compare across a large number of countries, particularly in light of the scarce data on global prevalence of flavored cigarette use^12^. Moreover, our study offers new insight on which country-level factors may be in part driving growth of FCCs and MNCCs and/or which country-level factors may be targeted by the tobacco industry in its marketing efforts.

## CONCLUSIONS

This study provides a global snapshot of the market landscape of flavor capsule cigarettes (FCCs) and menthol (non-capsule) cigarettes (MNCCs). We found that FCCs have experienced substantial growth, particularly among countries of the WHO Americas region and in upper middle-income countries, from 2010 to 2020. The overall market share of MNCCs slightly decreased, but remained high in many countries, with the highest shares in the WHO Western Pacific and Africa regions. By 2019, FCCs made up a larger proportion of the global cigarette market than MNCCs. Country-level factors associated with market shares of FCCs and/or MNCCs, such as smoking prevalence, unemployment rate, and urbanicity, found in this study, may be associated with popularity of these products in some countries versus others. Policymakers should be aware of these factors that may contribute to higher market shares of these products. Moreover, given that these country characteristics may also be indicative of tobacco industry’s marketing efforts and priorities, tobacco industry activity should also be closely monitored. Findings support the critical need for increased efforts to address flavors and innovative features used in tobacco products. These data can be used by advocates and policy makers to monitor and support implementation of measures to curb growth of flavored cigarettes. Given the scant global epidemiological data on prevalence of flavored tobacco products, future research should prioritize population-level studies for more comprehensive surveillance, particularly in priority countries and regions identified in this study.

## Data Availability

The data supporting this research are available from the following source: https://www.euromonitor.com/. Imperial College London subscribes to Euromonitor Passport and the data were accessed through the Imperial College London library portal.

## References

[1] World Health Organization (2021). WHO Report on the Global Tobacco Epidemic 2021: Adressing New and Emerging Products.

[2] World Health Organizaton (2011). WHO Framework Convention on Tobacco Control: Guidelines for Implementation Article 5.3; Article 8; Articles 9 and 10; Article 11; Article 12; Article 13; Article 14.

[3] Chung-Hall J, Craig L, Gravely S, Sansone N, Fong GT (2019). Impact of the WHO FCTC over the first decade: A global evidence review prepared for the Impact Assessment Expert Group. Tob Control.

[4] Gilmore AB (2012). Understanding the vector in order to plan effective tobacco control policies: an analysis of contemporary tobacco industry materials. Tob Control.

[5] World Health Organization (2016). Advisory Note: Banning Menthol in Tobacco Products.

[6] Villanti AC, Johnson AL, Glasser AM (2019). Association of Flavored Tobacco Use With Tobacco Initiation and Subsequent Use Among US Youth and Adults, 20132015. JAMA Netw Open.

[7] Nonnemaker J, Hersey J, Homsi G, Busey A, Allen J, Vallone D (2013). Initiation with menthol cigarettes and youth smoking uptake. Addiction.

[8] Lewis MJ, Wackowski O (2006). Dealing with an innovative industry: a look at flavored cigarettes promoted by mainstream brands. Am J Public Health.

[9] Kreslake JM, Wayne GF, Connolly GN (2008). The menthol smoker: Tobacco industry research on consumer sensory perception of menthol cigarettes and its role in smoking behavior. Nicotine Tob Res.

[10] Zare S, Nemati M, Zheng Y (2018). A systematic review of consumer preference for e-cigarette attributes: Flavor, nicotine strength, and type. PLoS One.

[11] Thrasher JF, Islam F, Barnoya J, Mejia R, Valenzuela MT, Chaloupka FJ (2017). Market share for flavour capsule cigarettes is quickly growing, especially in Latin America. Tob Control.

[12] Kyriakos CN, Zatoński MZ, Filippidis FT (2022). Marketing of flavour capsule cigarettes: a systematic review. Tob Control.

[13] Miller Lo EJ, Young WJ, Ganz O, Talbot EM, O'Connor RJ, Delnevo CD (2022). Trends in Overall and Menthol Market Shares of Leading Cigarette Brands in the USA: 2014-2019. Int J Environ Res Public Health.

[14] Miller Lo EJ, Giovenco DP, Wackowski OA, Harrell MB, Perry CL, Delnevo CD (2017). The Cigarette and Smokeless Tobacco Markets in Texas Relative to the United States. Tob Regul Sci.

[15] Kuiper NM, Gammon D, Loomis B (2018). Trends in Sales of Flavored and Menthol Tobacco Products in the United States During 2011-2015. Nicotine Tob Res.

[16] King B, White V, Balmford J, Cooper J, Borland R (2012). The decline of menthol cigarette smoking in Australia, 1980-2008. Nicotine Tob Res.

[17] van der Eijk Y, Lee JK, M Ling P (2019). How Menthol Is Key to the Tobacco Industry's Strategy of Recruiting and Retaining Young Smokers in Singapore. J Adolesc Health.

[18] Giovino GA, Sidney S, Gfroerer JC (2004). Epidemiology of menthol cigarette use. Nicotine Tob Res.

[19] Moodie C, Thrasher JF, Cho YJ, Barnoya J, Chaloupka FJ (2019). Flavour capsule cigarettes continue to experience strong global growth. Tob Control.

[20] Hiscock R, Silver K, Zatoński M, Gilmore AB (2020). Tobacco industry tactics to circumvent and undermine the menthol cigarette ban in the UK. Tob Control.

[21] Zatoński M, Silver K, Plummer S, Hiscock R (2022). Menthol and flavored tobacco products in LMICs: A growing menace. Tob Induc Dis.

[22] Villanti AC, Mowery PD, Delnevo CD, Niaura RS, Abrams DB, Giovino GA (2016). Changes in the prevalence and correlates of menthol cigarette use in the USA, 2004–2014. Tob Control.

[23] Kyriakos CN, Zatoński MZ, Filippidis FT (2021). Flavour capsule cigarette use and perceptions: a systematic review. Tob Control.

[24] Zatoński M, Herbeć A, Zatoński W (2018). Characterising smokers of menthol and flavoured cigarettes, their attitudes towards tobacco regulation, and the anticipated impact of the Tobacco Products Directive on their smoking and quitting behaviours: The EUREST-PLUS ITC Europe Surveys. Tob Induc Dis.

[25] Kaleta D, Usidame B, Szosland-Fałtyn A, Makowiec-Dąbrowska T (2014). Use of flavoured cigarettes in Poland: data from the global adult tobacco survey (2009-2010). BMC Public Health.

[26] Lee YO, Glantz SA (2011). Menthol: putting the pieces together. Tob Control.

[27] La Torre G, Mipatrini D (2016). Country-level correlates of e-cigarette use in the European Union. Int J Public Health.

[28] Feliu A, Filippidis FT, Joossens L (2021). The association between tobacco control policy implementation and country-level socioeconomic factors in 31 European countries. Sci Rep.

[29] Passport Euromonitor International.

[30] Laverty AA, Millett C, Filippidis FT (2021). Associations between cigarette prices and consumption in Europe 2004-2014. Tob Control.

[31] Filippidis FT, Laverty AA, Hone T, Been JV, Millett C (2017). Association of Cigarette Price Differentials With Infant Mortality in 23 European Union Countries. JAMA Pediatr.

[32] Kyriakos CN, Ahmad A, Chang K, Filippidis FT (2021). Price differentials of tobacco products: A cross-sectional analysis of 79 countries from the six WHO regions. Tob Induc Dis.

[33] World Health Organization Country groupings.

[34] The World Bank World Bank Country and Lending Groups.

[35] International Monetary Fund (2021). World Economic Outlook Database April 2022.

[36] World Health Organization Tobacco Control.

[37] Dubray J, Schwartz R, Chaiton M, O’Connor S, Cohen JE (2015). The effect of MPOWER on smoking prevalence. Tob Control.

[38] Delnevo CD, Giovenco DP, Villanti AC Impact of menthol capsule cigarettes on menthol and non-menthol cigarette consumption in the USA, 2008–2020. Tob Control.

[39] Erinoso O, Clegg Smith K, Iacobelli M, Saraf S, Welding K, Cohen JE (2020). Global review of tobacco product flavour policies. Tob Control.

[40] World Health Organization (2017). Partial Guidelines for Implementation of Articles 9 and 10.

[41] Cruz TB, Wright LT, Crawford G (2010). The Menthol Marketing Mix: Targeted Promotions For Focus Communities in the United States. Nicotine Tob Res.

[42] Sóñora G, Reynales-Shigematsu LM, Barnoya J, Llorente B, Szklo AS, Thrasher JF (2022). Achievements, challenges, priorities and needs to address the current tobacco epidemic in Latin America. Tob Control.

[43] Kyriakos CN, Fong GT, de Abreu Perez C (2022). Brazilian smokers are ready for the ban on flavour additives in tobacco to be implemented. Prev Med.

[44] Oliveira da Silva AL, Bialous SA, Albertassi PGD, Arquete DADR, Fernandes AMMS, Moreira JC (2019). The taste of smoke: tobacco industry strategies to prevent the prohibition of additives in tobacco products in Brazil. Tob Control.

[45] Scollo M, Bayly M, White S, Lindorff K, Wakefield M (2018). Tobacco product developments in the Australian market in the 4 years following plain packaging. Tob Control.

[46] Moodie C, Angus K, Mitchell D, Critchlow N (2018). How tobacco companies in the United Kingdom prepared for, and responded to, standardised packaging of cigarettes and rolling tobacco. Tob Control.

[47] van der Eijk Y, Yang AY Tobacco industry marketing adaptations to Singapore plain packaging. Tob Control.

[48] Barnoya J, Monzon D, Pinetta J, Grilo G, Cohen JE New tobacco products, old advertising strategies: point-of-sale advertising in Guatemala. Tob Control.

[49] Institute for Global Tobacco Control (2017). Technical Report on Flavored Cigarettes at the Point-of-Sale in Latin America: Availability and Marketing around Primary and Secondary Schools in Five Countries.

[50] Paz Ballesteros WC, Pérez Hernández R, Thrasher LaFontaine JF (2018). Tobacco retail and publicity at points of sale (PoS) around schools in three major cities in Mexico (2014-2016). Tob Induc Dis.

[51] Llambi L, Minacapilli M, Barros M, Parodi C, Peluffo VG (2021). Cigarette flavours and design features available near schools before plain packaging implementation in Uruguay. Arch Community Med Public Health.

[52] Gallagher A, Gilmore A (2020). Euromonitor International now accepts tobacco industry funding: a win for PMI at the expense of research on the tobacco industry. Tobacco Control blog.

[53] Gallagher AWA, Evans-Reeves KA, Hatchard JL, Gilmore AB (2019). Tobacco industry data on illicit tobacco trade: a systematic review of existing assessments. Tob Control.

