# Supplementary material for: Global market trends of flavor capsule cigarettes and menthol (non-capsule) cigarettes: An ecological analysis using commercial data across 78 countries, 2010–2020
Source: Tob Induc Dis. 2022 Oct 10;20:85. doi: 10.18332/tid/153974 (PMC9549585; doi:10.18332/tid/153974)
Supplement: Supplementary file 1 [file TID-20-85-s1.pdf]

SUPPLEMENTARY MATERIAL

Supplementary Figure 1. Average annual growth rates[1] from 2010 to 2020 by % market share in 2020[2] for flavour capsule cigarettes and menthol cigarettes by country income level, Euromonitor Passport

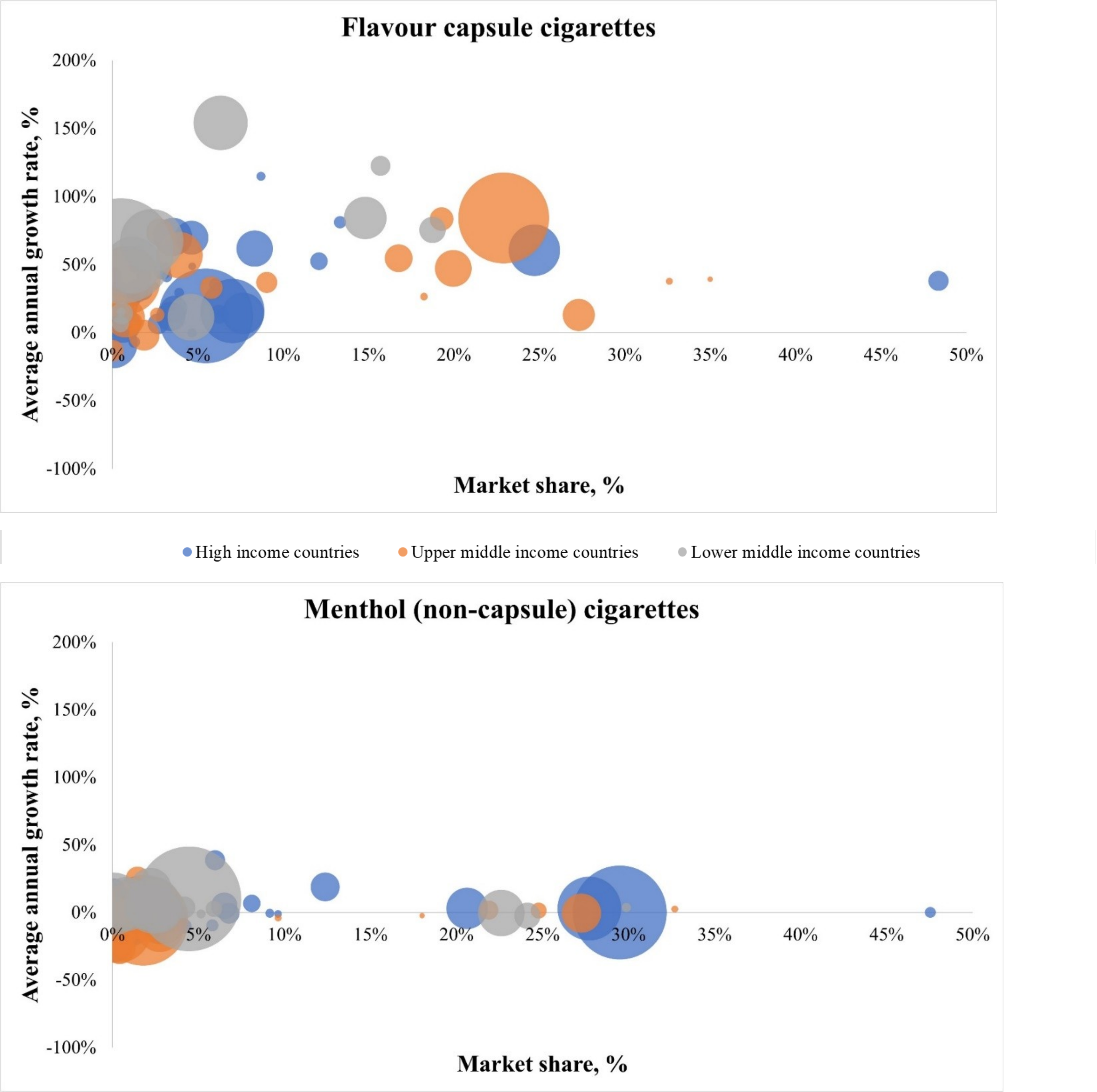

Bubble size corresponds to total cigarette retail size volume; For figure visualisation purposes, China was set to twice the size of Indonesia, the country with the second largest cigarette retail volume (in actuality, China's volume is 8.6 times that of Indonesia)

Flavour capsule cigarettes: N=21 countries in 2010 and 64 in 2020; Menthol cigarettes: N=78 in 2010 to N=74 in 2020

[1] Annual growth rates >300% capped at 300% to avoid meaningless values resulting from an increase over a very small market share
[2] Or latest available year

© 2022 Kyriakos C.N. et al.
